# Mobile genetic element-driven genomic changes in a community-associated methicillin-resistant Staphylococcus aureus clone during its transmission in a regional community outbreak in Japan

**DOI:** 10.1099/mgen.0.001272

**Published:** 2024-07-17

**Authors:** Katsuyuki Katahira, Yasuhiro Gotoh, Kentaro Kasama, Dai Yoshimura, Takehiko Itoh, Chieko Shimauchi, Akihiko Tajiri, Tetsuya Hayashi

**Affiliations:** 1 Department of Bacteriology, Graduate School of Medical Sciences, Kyushu University, Higashi-ku, Fukuoka 812-8582, Japan; 2 Department of Respiratory Medicine, Graduate School of Medical Sciences, Kyushu University, Higashi-ku, Fukuoka 812-8582, Japan; 3 Department of Respiratory Medicine, NHO Omuta Hospital, Tachibana, Omuta City 837-0911, Japan; 4 Graduate School of Bioscience and Biotechnology, Tokyo Institute of Technology, Meguro-ku, Tokyo 152-8550, Japan; 5 Department of Nursing Humanics I, Miyazaki Prefectural Nursing University, Manabino, Miyazaki 880-0929, Japan; 6 Tajiri Dermatology Clinic, Kiyotake, Miyazaki 889-1067, Japan

**Keywords:** community-associated *Staphylococcus aureus*, genomic change, mobile genetic element, outbreak, whole-genome sequencing analysis

## Abstract

Community-associated methicillin-resistant *Staphylococcus aureus* (CA-MRSA) infections are now a public health concern in both community and healthcare settings worldwide. We previously identified a suspected case of a maternity clinic-centred outbreak of CA-MRSA skin infection in a regional community in Japan by PFGE-based analysis. In this study, we performed genome sequence-based analyses of 151 CA-MRSA isolates, which included not only outbreak-related isolates that we previously defined based on identical or similar PFGE patterns but also other isolates obtained during the same period in the same region. Our analysis accurately defined 133 isolates as outbreak-related isolates, collectively called the TDC clone. They belonged to a CA-MRSA lineage in clonal complex (CC) 30, known as the South West Pacific (SWP) clone. A high-resolution phylogenetic analysis of these isolates combined with their epidemiological data revealed that the TDC clone was already present and circulating in the region before the outbreak was recognized, and only the isolates belonging to two sublineages (named SL4 and SL5) were directly involved in the outbreak. Long persistence in patients/carriers and frequent intrahousehold transmission of the TDC clone were also revealed by this analysis. Moreover, by systematic analyses of the genome changes that occurred in this CA-MRSA clone during transmission in the community, we revealed that most variations were associated with mobile genetic elements (MGEs). Variant PFGE types were generated by alterations of prophages and genomic islands or insertion sequence (IS)-mediated insertion of a plasmid or a sequence of unknown origin. Dynamic changes in plasmid content, which were linked to changes in antimicrobial resistance profiles in specific isolates, were generated by frequent gain and loss of plasmids, most of which were self-transmissible or mobilizable. The introduction of IS*256* by a plasmid (named pTDC02) into sublineage SL5 led to SL5-specific amplification of IS*256,* and amplified IS*256* copies were involved in some of the structural changes of chromosomes and plasmids and generated variations in the repertoire of virulence-related genes in limited isolates. These data revealed how CA-MRSA genomes change during transmission in the community and how MGEs are involved in this process.

**Impact Statement**

Genome sequencing is now widely applied to investigations of various pathogen outbreaks due to its high resolution, ability to successfully identify outbreaks and ability to provide a wide range of valuable information, such as information on strain transmission during each outbreak. Although the contents of mobile genetic elements (MGEs) can change during outbreaks, systematic analyses of the full repertoire of MGEs (the mobilome) and its alteration during outbreaks have rarely been conducted. Here, we present the results of a genome-sequencing analysis of a suspected case of community-associated methicillin-resistant *Staphylococcus aureus* (CA-MRSA) infection that was previously identified by PFGE-based analysis. Our analysis accurately defined outbreak-related isolates and their transmission in the community. More importantly, systematic analyses of the variations in genome structure and sequence among these isolates revealed how CA-MRSA genomes changed during transmission in the community and the key roles of MGEs, such as prophages, genomic islands, insertion sequences (ISs) and plasmids, in this process.

## Data Summary

The raw sequences obtained in this study have been deposited in GenBank/EMBL/DDBJ under the BioProject accession number PRJDB10537 and the Sequence Read Archive (SRA) accession numbers DRR250723 to DRR250873, DRR253282 and DRR505234.

## Introduction

Methicillin-resistant *Staphylococcus aureus* (MRSA) infection is a major public health concern in both healthcare and community settings worldwide [1,2]. Originally, it was restricted to healthcare settings, but since the mid-1990s, MRSA clones, so-called community-associated MRSA (CA-MRSA), which have no epidemiological links to healthcare settings and can cause diseases in otherwise healthy individuals, have emerged in different geographic regions, and some clones have spread globally. The most frequent clinical manifestations of CA-MRSA are purulent skin and soft tissue infection (SSTI), which often represent recurrent and/or chronic infections, but severe, invasive diseases such as necrotizing pneumonia and sepsis can also occur [1,3, 4]. While CA-MRSA emerged from multiple *S. aureus* lineages defined by different sequence types (STs), such as ST1, ST8, ST30, ST59 and ST80, they share several phenotypic and genetic traits distinct from those of traditional healthcare-associated MRSA (HA-MRSA): resistance to fewer non-beta-lactams, carriage of the staphylococcal cassette chromosome *mec* (SCC*mec*) classified as type IV or V, and frequent production of Panton–Valentine leucocidin (PVL), a preforming toxin encoded by the *lukSF* genes located in prophages [4]. However, the definition of CA-MRSA is no longer strict because the transmission of CA-MRSA to healthcare settings is increasingly reported [5,6].

Similar to many other bacteria, mobile genetic elements (MGEs), such as plasmids, bacteriophages, transposons (Tns)/insertion sequences (ISs), and some genomic islands, played important roles in the evolution of *S. aureus* populations by mediating horizontal transfer of genes for virulence, antimicrobial resistance, and other properties [7,10]. For the emergence of MRSA lineages, various MGEs, including SCC*mec* and PVL-encoding bacteriophages mentioned above, have played crucial roles. Moreover, it has been reported that gain and loss of MGEs occur frequently, generating the variation in MGE repertoire in single MASA lineages [11] and even in colonizing MRSA populations colonizing in single hosts [12].

We previously identified a cluster of skin infections due to CA-MRSA in a region of Miyazaki Prefecture, Japan [13]. The cluster was first recognized by a sudden increase in the number of cases of skin MRSA infections, such as furuncles and carbuncles, among patients who visited a dermatology clinic (referred to as the D clinic) in 2001. As many of these patients were women who gave birth in a maternity clinic in the same region (referred to as the M clinic) and other members of their households, such as their children, husbands and parents, we suspected the occurrence of an M clinic-centred outbreak of CA-MRSA infection. Thus, we screened MRSA carriers among the staff of the M clinic and performed PFGE analysis of the MRSA isolates. We identified staff with nasal MRSA in the screening, and most MRSA isolates in the D clinic and the isolates from staff of the M clinic showed similar SmaI-digestion patterns (a less than four-band difference; we referred to this type as PFGE type 1 and recognized eight variations named types 1a-1h). After decolonization was performed twice in the M clinic at the end of 2001, the isolation of PFGE type 1 MRSA from patients with skin infections in the D clinic gradually decreased but continued for more than 7 years. Finally, we identified 130 PFGE type 1 isolates, which included multiple isolates from the same individuals or households. All the strains produced type 4 coagulase and were PCR-negative for enterotoxins A, B, C, and D and toxic shock syndrome toxin-1 genes. However, all but one strain contained the gene-encoding PVL. Based on these results, we concluded that an M clinic-centred outbreak of CA-MRSA occurred and continued for more than 7 years in the regional community. However, the 130 isolates included a notable number of PFGE type 1 isolates (27/130) with no apparent epidemiological links to the M clinic. Several isolates with epidemiological links to the M clinic but with different PFGE patterns were also isolated during the outbreak period. The resolution of PFGE is too low to understand the genetic relatedness of these isolates to epidemiological link-positive PFGE type 1 isolates, and whole-genome sequence (WGS) information is necessary [14,15]. Moreover, WGS analysis of outbreak-related isolates could provide a good opportunity to analyse how the CA-MRSA genome changes during transmission in the community. Therefore, in this study, we performed WGS analysis of MRSA isolates obtained during the outbreak period. The results obtained by integrated analyses of genomic and epidemiological data revealed accurate genetic relationships between the MRSA isolates, the genomic features of the outbreak clone, and dynamic genomic changes of this clone during transmission in the community, which were driven by MGEs.

## Methods

### Bacterial isolates sequenced in this study

The MRSA isolates sequenced in this study (*n*=151) are listed in Table S1 (available in the online version of this article), along with their sequencing statistics, STs, clonal complexes (CCs), and accession numbers. The epidemiological link to the M clinic, date of isolation, clinical information, body site of isolation, PFGE type, presence of PVL, and staphylococcal protein A (spa) and SCC*mec* types of the 133 outbreak-related isolates defined in this study are provided in Table S2. These isolates were distinguished by unique identifiers (IDs) indicating case numbers (Cxxx), household numbers (Hxx), and epidemiological links to the M clinic (B, person who gave birth at the M clinic; R, household member of B; S, staff or in/outpatient of the M clinic; and N, unknown). When multiple isolates were obtained from the same patient at different time points, these isolates were indicated by the addition of ‘a’, ‘b’ or ‘c’ at the end of the isolate ID according to the order of isolation. Isolates from the nasal cavity were identified by adding ‘-n’ to the end of the isolate ID.

### Genome sequencing and assembly

Genomic DNA was extracted and purified from overnight cultures grown at 37 °C in 1 ml of tryptic soy broth [TSB; Becton, Dickinson and Company (BD)] using the Maxwell Cell DNA Purification Kit with automatic DNA extraction by a Maxwell 16 MDx Instrument (Promega) according to the manufacturer’s instructions. Sequencing libraries were prepared using the KAPA HyperPlus Library Preparation Kit for Illumina (KAPA Biosystems) and sequenced using Illumina MiSeq to generate paired-end reads (301 bp × 2). Genomic DNA for long-read sequencing was purified using the QIAGEN Genomic-tip 500/G Kit (Qiagen). Long-read sequencing libraries were prepared without fragmentation using a 1D ligation sequencing kit [SQK-LSK108, Oxford Nanopore Technologies (ONT)] according to the manufacturer’s instructions and sequenced on the ONT MinION platform using an R9.4 flow cell (FLO-MIN106, ONT). ONT reads were base-called using Albacore v2.2.5 (ONT). Genome assembly of Illumina short reads was performed using Platanus v1.2.4 (with the parameter ‘-u 0’) [16]. The complete genome sequences of isolates C005H04R and C089H64Rb were obtained by hybrid assembly of Illumina and ONT reads using Unicycler v0.4.7 [17].

### Gene annotation and IS and prophage search

Gene annotation and searches for ISs and prophages of the C005H04R genome, which was used as a reference in this study, were carried out using dfast v1.2.6 [18], IS Finder [19], and phaster [20], respectively, followed by manual curation using IMC-GE software (In Silico Biology). DNA plotter v18.1.0 [21] was used to construct a circular map of the C005H04R chromosome. The copy numbers of ISs in draft genomes were estimated by ISseeker v1.1 [22] with default parameters except that the sequences of IS*256*, IS*Sau1*, IS*Sau2*, IS*Sau3* and IS*431mec* found in the C005H04R genome were used as references with a >90 % identity threshold. The results of the copy number comparison were visualized using ggplot2 in R v4.2.2.

### WGS-based phylogenetic and temporal analyses

We performed two types of WGS-based phylogenetic analyses: first, to determine the phylogenetic position of the CC30 MRSA isolates identified in this study in the global CC30 population, and second, to obtain an accurate and higher-resolution phylogenetic tree of the outbreak-related isolates.

In the first analysis, the assembly data of CC30 *S. aureus* genomes were obtained from the National Center for Biotechnology Information (NCBI) RefSeq Genomes database (accessed on 9 November 2020) and quality-filtered using CheckM v1.1.10 [23] with a cutoff of >99 % completeness and <1 % contamination. Genome sequences of the outbreak-related isolates examined in this study (*n*=132, in addition to the C005H04R reference genome) and those of the CC30 strains obtained from the NCBI (*n*=452) and four strains (WBG10049, 58–424, A017934/97, and MN8; used as phylogenetic markers) were aligned to the finished chromosome sequence of C005H04R, which was masked by the sequences of SCC*mec*, ISs, prophages, and *rrn* operons. Sequence alignment was performed in MUMmer v3.1 [24] with cutoff thresholds of 96 % for sequence identity and 2 000 bp for alignment length to identify SNP sites in the core-genome sequence (1 614 844 bp). To obtain high-confidence SNPs, we selected SNPs in >500 bp sequences shared by all strains, and those residing within 100 bp of alignment boundaries or within a 5 bp distance of any insertion/deletion (InDel) sites were excluded [25]. Recombinogenic SNP sites were further removed by Gubbins v2.2.0 [26]. Using the final SNP set (*n*=23,157), a maximum-likelihood (ML) phylogenetic tree was constructed by RAxML-NG v1.0.1 [27] with the GTR model of nucleotide substitution, which was selected by ModelTest-NG v0.1.6 [28], and 1000 bootstraps. The tree was displayed and annotated using iTOL v6 [29]. A list of CC30 *S. aureus* genomes obtained from the NCBI and used for this analysis (*n*=456) is provided in Table S3.

In the second analysis, only the genome sequences of the 132 outbreak-related isolates were aligned to the C005H04R chromosome sequence using MUMmer v3.1 with cutoff thresholds of 99 % for sequence identity and 2 000 bp for alignment length to obtain the core-genome sequence of 133 outbreak-related isolates (2 623 370 bp), and high-confidence core-genome SNPs were identified as described above. After removing SNPs in repeat sequences (ISs, *rrn* operons, and sequences identified by self-alignment of the C005H04R chromosome by blastn with cutoff thresholds of 95 % for identity and 50 bp for alignment length) and recombinogenic SNPs identified by Gubbins v2.2.0, the remaining SNP sites in each strain were checked and corrected by Illumina read-mapping using Burrows–Wheeler Aligner (BWA) [30] in BactSNP software v1.1.0 [31] with the default threshold setting (allele frequency >0.9, coverage depth >10). Using the 478 SNP sites identified, a phylogenetic tree was constructed by the neighbor-joining (NJ) method and the *p*-distance model with 1000 bootstrap replications in mega v7 [32]. The tree was displayed and annotated using iTOL v6, and the SNP sites identified were annotated using SnpEff v4.3 [33]. Pairwise SNP distance matrices were obtained using snp-dists v0.7.0 (https://github.com/tseemann/snp-dists).

Temporal analysis of the outbreak-related isolates was performed by TempEst v1.5.1 [34]. For this analysis, we first identified groups of isolates sharing an identical core genome and selected one isolate from the earliest isolation date in each group. Using the genome sequences of the 105 selected isolates, an ML tree was constructed by RAxML-NG v1.0.1 with the TPM2uf+I model selected by ModelTest-NG v0.1.6 and subjected to TempEst analysis.

The pairwise SNP distance matrices of six ST59/SCC*mec* Vb/spa t437 isolates were obtained using the same method as described above except that high coverage contigs (>2 times higher than the average of >1 kb contigs) were excluded from the analysis because they were potentially derived from MGEs.

### Molecular typing

The STs and CCs of the isolates were determined by mlst v2.19.0 [35] with the PubMLST database for *S. aureus* [36]. SCC*mec* and spa typing was carried out by SCC*mec*Finder v1.2 [37,38] and spaTyper v1.0 [39], respectively, with default settings.

### Analysis of the variation in genome structure between different PFGE types

To analyse the variation in chromosome structures associated with each PFGE type (types 1 a-1h) in the outbreak-related isolates, we first identified 29 SmaI fragments of the C005H04R chromosome (PFGE type 1a) by *in silico* digestion using Genetyx-Mac (Genetyx). The 29 fragments were aligned with contigs (>1 kb) of the draft genomes representing each PFGE type using GenomeMatcher v3.04 [40] to identify the sequences responsible for the difference in the PFGE pattern (Fig. S1).

### Plasmid analysis

To identify plasmid sequences in draft genomes, each genome was reassembled by platanus_B v1.1.0 [41], which identifies potentially circular sequences, and ‘circular candidate’ contigs were selected. In addition, each genome was searched by blastn using the C005H04R chromosome sequence as a query with the following parameters: 40 % minimum coverage and 80 % minimum identity. No hit contigs were identified as potentially plasmid-related sequences and analysed by manual inspection of their annotations via dfast v1.2.6 and Prokka v1.2 [42], blastn search (https://blast.ncbi.nlm.nih.gov/Blast.cgi) against the nr/nt database of the NCBI, and plasmid replicon search in PlasmidFinder v2.1 [43] with filtering at a ≥95 % nucleotide sequence identity and ≥60 % coverage. Through these analyses, we identified 13 plasmids and plasmid-like sequences corresponding to pTDC01A, pTDC02, pTDC03A, pTDC03B, pTDC04A, pTDC04B, pTDC04C, pTDC04D, pTDC05, pTDC06, pTDC07A, pTDC07B, and pTDC08 in the final plasmid set. The circularity and contig end sequence of the plasmid-like sequences were confirmed by PCR and amplicon sequencing. The 13 plasmids were classified into eight types (named pTDC01-pTDC08) according to their replicon types determined by PlasmidFinder v2.1 with default settings and sequence similarity. The distribution of these plasmids and their genomic variations among the outbreak-related isolates were analysed by read mapping using BWA with default settings and the sequences of eight plasmids as references (pTDC01A, pTDC02, pTDC03A, pTDC04A, pTDC05, pTDC06, pTDC07A, and pTDC08 in the final plasmid set). The results of read mapping for each genome were manually inspected using Integrative Genomics Viewer (IGV). When unmapped regions were found, the sequences of these regions in the assembled data (contig sequences) were analysed to determine their sequence variations. As unmapped regions were not assembled in several pTDC01-related plasmids, the sequences of these regions were determined by PCR and amplicon sequencing. By this analysis, we identified two variants of pTDC01 (pTDC01B and pTDC01C). The sequences of these two variants were further confirmed by Illumina read mapping. When a sequence variation was a simple InDel or IS insertion, the plasmid was regarded as a small variant and indicated by the addition of ‘_v’.

The pWBG749-like and pSK41-like *oriT* mimic sequences in the eight plasmids found among the 133 isolates were searched by blastn using the pWBG749 *oriT* and pSK41 *oriT* sequences [44] as queries. The distribution of plasmids related to the eight plasmids found in the outbreak-related isolates in the abovementioned global CC30 *S. aureus* genome set was analysed by blastn search using the replicon sequences in each plasmid as queries with cutoff thresholds of 99 % for minimum coverage and 99 % for minimum sequence identity. Plasmids highly similar to the eight plasmids found in the outbreak-related isolates were also searched against the nr/nt database using Megablast with cutoff thresholds of 80 % for minimum coverage and 95 % for sequence identity.

### Antimicrobial susceptibility testing and antimicrobial resistance (AMR) gene analysis

Antimicrobial susceptibility testing was carried out by the microdilution method in accordance with the Clinical and Laboratory Standards Institute (CLSI) guidelines. *S. aureus* strain ATCC29213 was used for quality control. Thirteen antimicrobial agents were used: penicillin G (Sigma‒Aldrich), oxacillin (Wako), cefoxitin (Honeywell Fluka), minocyclcin (Pfizer), gentamicin (Sigma‒Aldrich), kanamycin (Meiji Seika Pharma Co.), erythromycin (Sigma‒Aldrich), fosfomycin (Sigma‒Aldrich), sulfamethoxazole-trimethoprim (Wako), levofloxacin (Daiichi Sankyo), vancomycin (Sigma‒Aldrich), teicoplanin (Sanofi), and rifampicin (Nacalai Tesque).

Acquired AMR genes in the genomes of the outbreak-related isolates were identified by the Short Read Sequence Typing for Bacterial Pathogens (srst2) programme v0.2.0 (maximum divergence; 1  %) [45] using the Comprehensive Antibiotic Resistance Database (card) v2.0.0 [46]. We added the *blaZ* gene found in outbreak-related isolates to the database because its sequence showed >1 % divergence from the reference *blaZ* gene sequence in the CARD.

### Conjugation experiments

Spontaneous rifampicin (RIF)-resistant mutants of the C001F01B and C006F05R isolates carrying pTDC01A and pTDC02, respectively, were obtained by serial passage for 7 days using Muller–Hinton II broth (BD) supplemented with a subminimum inhibitory concentration (MIC) of RIF. These mutants showed a 212-fold increase in the MIC compared to the original MIC (0.001 µg ml^−1^). Conjugation experiments were carried out using isolate C082F60S-n, which carried pTDC03A, as a donor strain and the RIF-resistant derivatives of C001F01B and C006F05R as recipients via the filter-mating method [47] as follows. One millilitre of the overnight cultures of the donor and recipient cells grown in TSB with shaking at 37 °C were diluted with fresh TSB to an optical density at 600 nm (OD_600_) of 1, and the donor and recipient cells (0.5 ml each) were suspended in 50 ml of PBS. The suspension was filtered through a sterile analytical filter unit (0.2 µm pore size; Thermo Fisher). The filter membrane was retrieved from the unit and placed on a TSB agar plate. After incubation at 37 °C for 20–24 h, the membrane was transferred to a 50 ml tube containing 10 ml of PBS, and bacterial cells on the membrane were suspended in PBS by vortexing. A serial 10-fold dilution of the bacterial cell suspension was prepared with TSB, and 100 µl of the 10^−1^–10^−3^ diluted samples was inoculated onto TSB agar plates supplemented with 4.8 µg ml^−1^ RIF and 64 µg ml^−1^ kanamycin (KM) to select transconjugants. Similarly, 100 µl of the 10^−5^–10^−7^ diluted cell suspension was inoculated onto TSB agar plates supplemented with 4.8 µg ml^−1^ RIF to select recipients. After incubation at 37 °C for 18–24 h, the numbers of colonies on both plates were counted, and conjugation efficiency was calculated by dividing the number of transconjugants by that of recipients. Finally, the presence of the plasmids pTDC03A, pTDC01A and pTDC02 in each transconjugant was analysed by colony PCR using primers specific to each plasmid (Table S4).

### Virulence gene analysis

Virulence-related genes of isolate C005H04R were identified by SRST2 (maximum divergence, 1  %) using the Virulence Factor Database (VFDB) dataset B downloaded on 22 August 2020 [48]. Using the sequences of identified genes (*n*=83) as queries, the presence or absence of these genes in draft genomes was examined by blastn v2.4.0+ (cutoff, 100 % sequence identity and coverage). When a gene was defined as ‘absent’ in an isolate, the presence/absence of this gene was determined by aligning the query sequence to the relevant contig using ClustalW (https://www.genome.jp/tools-bin/clustalw) and/or by mapping the Illumina reads of this isolate to the C005H04R reference sequence using SRST2. When some sequence variations (SNPs and InDels) were detected, the variations were further examined by amino acid sequence comparison (synonymous or nonsynonymous SNPs, positions and sizes of InDels, and occurrence of frameshifts). Genes that were truncated by the introduction of a stop codon were regarded as inactivated. When an intramolecular repeat-associated in-frame InDel was found, this gene was regarded as functional. When a repeat-containing gene was split into different contigs in a genome, the presence or absence of this gene was determined by read mapping using SRST2.

## Results and discussion

### Defining outbreak-related CA-MRSA isolates and their phylogenetic positions in the global CC30 population

**Fig. 1.**
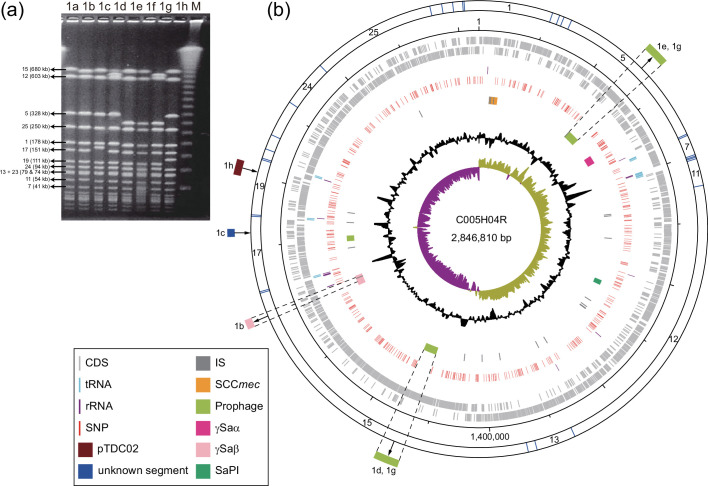
PFGE patterns of the TDC clone isolates and a circular map of the chromosome of isolate C005H04R. (**a**) SmaI-digestion patterns (PFGE type 1 a to 1 h) of the TDC clone isolates. The figure was taken from our previous article [7] with modifications. M, 100 bp DNA ladder. (**b**) From outside to inside represents the scale in bp, coding sequences (CDSs), tRNA and rRNA genes, the location of SNPs found in 133 TDC clone isolates analysed in this study, and mobile genetic elements, G+C content, and GC skew. The genetic changes responsible for the difference in PFGE band patterns (relative to type 1a) are also indicated.

To accurately define outbreak-related isolates, we sequenced 151 MRSA isolates obtained in our previous study [13] (Table S1). They included not only the 130 isolates that we defined as being related to the M clinic-centred outbreak based on the PFGE pattern (PFGE type 1 and its variants showing fewer than four band differences; Fig. 1a) but also 21 isolates that were isolated from patients who visited the D clinic with skin infections during the same period that outbreak-related isolates were obtained (2001–2007). Of the 151 isolates, the complete genome sequence of the earliest isolate, C005H04R, was determined by a hybrid sequence strategy to obtain the reference sequence for downstream analyses. The genome of an additional isolate (C089H64Rb) was also closed to confirm its PFGE pattern (originally assigned to type 1f but reassigned to type 1e).

Sequence typing of these 151 isolates via WGS revealed that of the 151 isolates, 133 belonged to CC30, 130 to ST30 and 3 to ST4216 (a single-locus variant of ST30) (Table S1). The SCC*mec* and spa types of these 133 isolates were a variant of SCC*mec* IVc (containing IS*256* and IS*Sau1* instead of Tn*4001* in the J3 region) and spa-type t019, respectively. In addition, the *lukSF* genes were detected in all but two isolates. These findings suggested the close genetic relatedness of the 133 isolates. While 130 of the 133 isolates were assigned to PFGE type 1 in our previous analysis, three were assigned to other types. However, re-examination of their PFGE patterns revealed that the PFGE types of these three isolates (C092H66N, type 13; C093H67N, type 17; and C029H20Rb, type 24) were incorrectly assigned due to typing errors during manual inspection of band patterns.

**Fig. 2.**
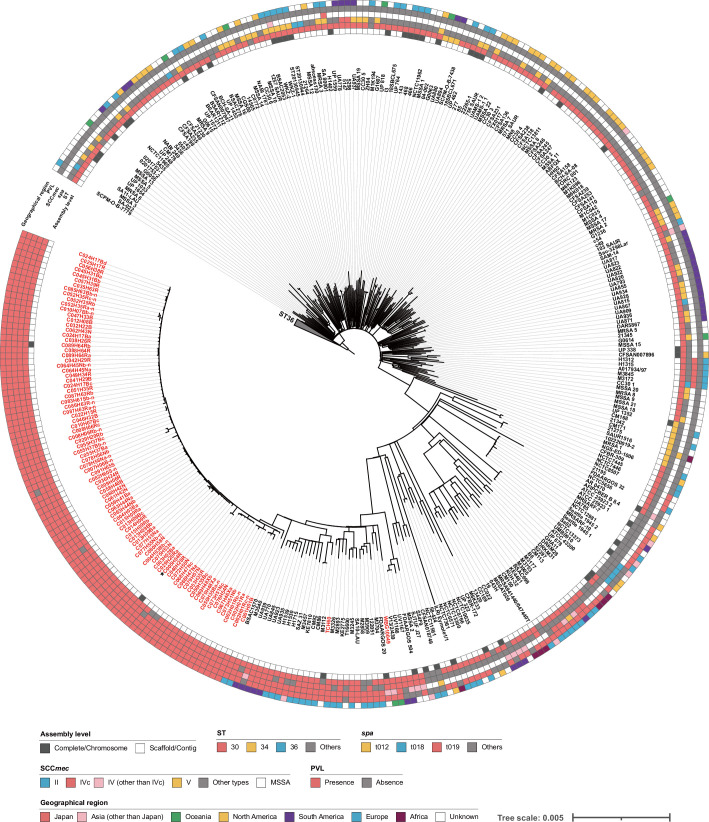
Phylogenetic position of the TDC clone in the global population of *S. aureus* CC30 strains. The ML tree constructed based on the 23 157 SNPs in the 1 614 844 bp core-genome sequence of 589 strains/isolates is shown along with strain information. The genome sequences of 456 of the 589 strains/isolates were obtained from the NCBI database. In this tree, strains belonging to ST36 were collapsed. TDC clone isolates and two strains known to represent the SWP clone are indicated in red. The C005H04R isolate, the genome of which was used as the reference, is indicated by an asterisk.

To examine the genetic relationships between the 133 CC30 isolates and their phylogenetic positions in the global population of CC30 *S. aureus* strains, we performed a WGS-based phylogenetic analysis of the 133 isolates along with 453 publicly available CC30 genomes (listed in Table S3). As shown in Fig. 2, the 133 isolates formed a tight cluster in a clade composed of mostly ST30/spa t019/SCC*mec* IV/PVL-positive strains, which included TCH60 and WBG10049, representative strains of a CA-MRSA lineage known as the ST30-MRSA IV or South West Pacific (SWP) clone [49,50]. This result indicated the clonal nature of the 133 isolates. In addition, this analysis revealed that the 133 isolates belonged to the ST30-MRSA IV/SWP clone, one of the globally disseminated CA-MRSA lineages [4,51], and the strain BSAC1570 isolated in England in 2007 [52] was most closely related to the 133 isolates.

Based on these results, we redefined the 133 isolates as outbreak-related isolates and they were collectively called the TDC (Tajiri Dermatology Clinic) clone and analysed in more detail. These strains were isolated from 93 patients/carriers and included many isolates obtained from the same patients/carriers (70 isolates from 28 individuals; up to four isolates per individual) and from multiple members (up to four) of the same households (66 isolates from 18 households).

Notably, six of the 18 isolates other than the 133 CC30 isolates were PVL-positive (Table S1). The PFGE type was type 10, and all the strains were ST59/SCC*mec* Vb/spa t437 MRSA. Thus, they probably belong to the so-called Taiwan clone, a major epidemic CA-MRSA lineage in East Asia [53,54], indicating that this clone was also circulating in this region when the TDC clone caused the M clinic-centred outbreak. They were isolated from patients belonging to different households during the period between 2003 and 2008. In the pairwise SNP distance analysis, while that of one pair (C0104H78N and C105H79N both isolated in 2007) was 11, those of all their pairs were 32–139, which are higher than ‘25 whole-genome SNPs’, a proposed cutoff to rule out recent (within 6 months) person-to-person transmission [55].

### Genomic features of the earliest isolate, C005H04R

To understand the genomic features of the TDC clone, we analysed the complete genome sequence of the earliest isolate, C005H04R. As summarized in Table 1, its genome comprises a 2 864 810 bp chromosome and a 28 598 bp plasmid (named pTDC02; see later sections for the details of this plasmid). In addition to the variant of IVc SCC*mec* mentioned above, the C005H04R chromosome also contained the γSaα and γSaβ genomic islands (GIs), a variant of the *S. aureus* pathogenicity island SaPI, and three prophages (Table 1; see Table S5 for more details).

**Table 1.** General genomic features of the C005H04R isolate

Chromosome	
Length	2 864 810 bp
G+C content (%)	32.9
Protein-coding sequences	2673
**Ribosomal RNAs**	
16S rRNA	6
23S rRNA	6
5S rRNA	7
Transfer RNAs	62
Prophages	3 (incl. 1 remnant)
SaPItdc phage-related island	1
SCC*mec* IVc variant	1
**Genomic islands**	
γSaα	1
γSaβ	1
**Insertion sequences**	
IS*Sau1*	7 (incl. 1 remnant)
IS*Sau2*	2
IS*Sau3*	8 (incl. 2 remnants)
ΨIS*1272* (in SCC*mec*)	1 (incl. 1 remnant)
IS*256*	3
IS*431mec* (in SCC*mec*)	1

Plasmid (pTDC02)	
Length	28 598 bp
G+C content (%)	27.6
Protein-coding sequences	32
**Insertion sequence**	
IS*256*	1
**Transposon**	
Tn*552*	1

The genetic organization of the γSaα and γSaβ GIs of C005H04R were almost the same as those of the CC30/ST36 strain MRSA252 [56,57]. SaPI is a prophage-related mobilizable element widely distributed in *S. aureus* that often encodes superantigen/enterotoxin genes [7], but the SaPI of C005H04R (named SaPItdc) encodes no superantigen genes. Many SaPI elements almost identical to SaPItdc (>99 % sequence identity and 100 % length coverage) were found not only in CC30 but also in other *S. aureus* lineages, such as CC1, CC5, and CC8. Of the three prophages (named phiTDC1, phiTDC2 and phiTDC3), phiTDC1 and phiTDC2 were apparently intact prophages. While phiTDC1 did not carry any known virulence determinants, phiTDC2 carried *lukSF*, the genes which encode PVL, and almost identical prophages (>99 % sequence identity and >99 % length coverage) were found in several ST30 strains. phiTDC3 (19 kb in length) was a degraded variant of phiSa3 integrated into the *hlb* gene encoding β-haemolysin. Compared with the phiSa3 found in the strain MRSA252 (43 kb in length), a 1.8 kb region containing the *sea* gene for staphylococcal enterotoxin A and half of the prophage (24 kb) were deleted from phiTDC3 (Fig. S2a).

Sequence comparison of the entire chromosome of C005H04R with the finished chromosomes of two strains belonging to the SWP clone, TCH60 and FDAARGOS_20, isolated in the UK and the USA, respectively (see Fig. 2 for their phylogenetic positions in CC30 and Table S3 for their accession numbers), revealed that C005H04R and these two strains shared a highly conserved chromosome (Fig. S3) and that the two strains contained GIs and prophages nearly identical to those in C005H04R except for the lack of phiTDC1 and a variation in the structure of type IV SCC*mec* (Fig. S2b).

In addition to these GIs and prophages, C005H04R also contained six types of IS elements (23 copies in total) and one transposon, Tn*522,* in the plasmid pTDC02 (Table 1; see Table S5 for details).

### Genetic relationships between the 133 outbreak-related MRSA isolates and identification of sublineages involved in the outbreak

To accurately determine the genetic relationships between the 133 isolates and how they relate to the epidemiological data of each isolate, we performed a high-resolution phylogenetic analysis of the 133 isolates based on the 478 recombination-free SNPs identified on their 2 623 370 bp conserved chromosomal backbone (see Table S6 for the list of SNPs). The maximum pairwise SNP distance among the 133 isolates was 77, confirming their very high relatedness. As summarized in Table S7, the maximum within-individual SNP distance was 25 (in C002; isolation interval was 7 months), but most cases (24/28 individuals) were less than 10. The maximum within-household SNP distance was 41 (in H01; isolation interval was 21 months), but in others (23/24 households), pairwise SNP distances were less than 20, lower than ‘25 whole-genome SNPs’ mentioned above [55] (note that isolates from household H35 were divided into two groups, H35-1 and H35-2, as described later).

**Fig. 3.**
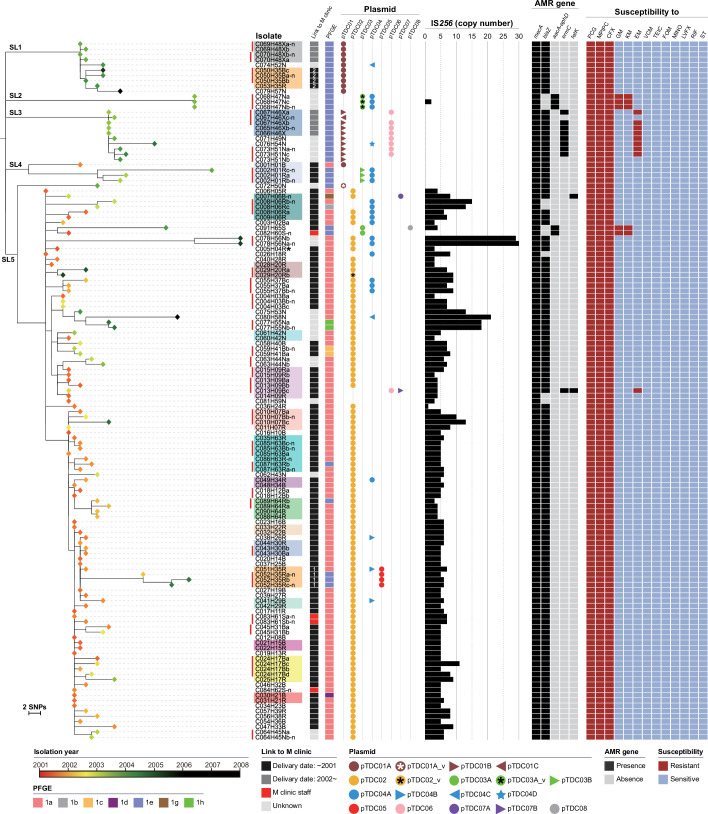
Relationships among the 133 TDC clone isolates. An NJ tree constructed based on 478 SNPs identified in the 2 623 370 bp core-genome sequence of 133 isolates is shown along with the information for each isolate: year of isolation (coloured rhombuses at the tips of the tree), epidemiological link to the M clinic, PFGE type, plasmid repertoire, copy number of IS*256*, AMR genes, and susceptibility to 13 antimicrobial agents. Isolates from the same individuals are indicated by red vertical lines, and isolates from the same households are indicated by colour shading. Five sublineages (SL1-SL5) defined for the TDC clone are indicated in the tree. The C005H04R isolate used as the reference is indicated by an asterisk.

As shown in Fig. 3, isolates obtained from the same individuals clustered together in all cases (*n*=28), irrespective of the site of isolation (affected skin site or nasal cavity). In several cases, the dates of the first and last isolations were notably separated, for example, more than 1 year in six cases (up to 33 months in case 10), indicating the long persistence of the TDC clone leading to the recurrent skin infections observed in these individuals. Similarly, isolates obtained from members of the same household clustered together in almost all cases (*n*=17), indicating frequent within-household transmission of the TDC clone, although actual transmission frequency was unknown because we did not employ a systematic sampling strategy. The only exception was the isolates from household H35; they formed two distantly related clusters (marked with ‘1’ and ‘2’ in the ‘Link to M clinic’ column in Fig. 3), indicating that the TDC clone invaded this household twice and caused within-household transmission independently. Importantly, the isolation periods of the TDC clone in many households were also considerably long—for example, more than 1 year in eight households (up to 41 months in household H20). This finding indicates the long persistence of the TDC clone in households, supporting the notion that households are primary reservoirs for MRSA in the community [58,59]. The ‘ping–pong’ transmissions in these households may have contributed to the prolonged persistence in household members [60].

Another interesting finding of this phylogenetic analysis (Fig. 3) was the presence of five distinct sublineages (named SL1–SL5). These sublineages appear to have been independently derived from a common ancestor in early 1997, as estimated from the result of the root-to-tip analysis (Fig. S4), although a caution is required for the interpretation of this result (a relatively low correlation coefficient), which was probably due to a short period of time to gain a fully reliable temporal signal. Importantly, however, only the isolates belonging to SL4 and SL5 had epidemiological links to the M clinic (links to childbirth at the M clinic before the end of 2001, when decolonization of clinic staffs was completed). The isolates from household H35 were originally regarded as epidemiologically linked because a member of H35 gave birth at the M clinic in 2001. However, the first isolation of the TDC clone in one cluster belonging to SL1 (indicated by ‘2’ in Fig. 3) occurred in February 2004, while that in the other cluster (in SL5, indicated by ‘1’ in Fig. 3) was isolated in January 2002. Thus, the ‘2’ cluster was most likely not linked to the M clinic. All other isolates belonging to SL1–SL3 were also isolated after April 2003. These findings indicate that the TDC clone was circulating in the community of this region before the M clinic-centred outbreak was recognized and that the SL4 and SL5 lineages were responsible for this outbreak. The presence of a notable number of isolates with no clear epidemiological links to the M clinic in SL5 likely represents further transmission of SL5 in the community from cases/households directly involved in the M clinic-centred outbreak.

### Genetic events underlying the changes in PFGE pattern

To analyse the variation in chromosome structures associated with each PFGE type (types 1a-1h) in the outbreak-related isolates, we first identified 29 SmaI fragments of the C005H04R chromosome (PFGE type 1a) by *in silico* digestion. The 29 fragments were aligned with contigs (>1 kb) of the draft genomes representing each PFGE type to identify the sequences responsible for the difference in the PFGE pattern (Fig. S1). As sequences responsible for the variation in PFGE type 1h could not be identified by this strategy, we analysed the results of Illumina read mapping to the relevant SmaI fragment of isolate C005H04R (fragment 19) and identified an IS*256* insertion in the type 1h genome. However, the observed difference in band size could not be explained by a simple insertion of IS*256*. Therefore, we hypothesized that IS*256*-mediated integration of pTDC02, which contained an IS*256* copy, occurred in the type 1h genome, and this hypothetical structure was proven by PCR using a set of primer pairs designed on the basis of the chromosome and pTDC02 sequences flanking IS*256* (Fig. S1).

The results of this analysis revealed that, while the PFGE type of all 133 isolates was type 1, there were seven variants (Fig. 1a; note that type 1f was reassigned to type 1e, as mentioned above). Mapping the variant type information to the phylogenetic tree revealed that all isolates belonging to the four SLs other than SL5 were type 1e except for one type 1a isolate in SL4 (Fig. 3). Most SL5 isolates were type 1a, but SL5 included isolates of minor variants (1b, 1c, 1d, 1g, and 1h) and several type 1e isolates. These type 1e isolates were sporadically distributed among type 1a isolates, suggesting that the 1a-to-1e change occurred on multiple occasions in SL5.

To understand the genetic events underlying the variations and alterations in the PFGE pattern that occurred in the TDC clone, we determined the chromosome regions and their structures responsible for each variation by aligning the contigs of isolates representing each variant type and mapping their Illumina reads to the finished chromosome of C005H04R (type 1a), which was followed by PCR examination in the case of type 1h (Fig. S1). As summarized in Fig. 1b, this analysis revealed not only that the 1a-to-1e shift was caused by the deletion of phiTDC1 but also that the genetic events underlying all other changes (the 1a-to-1b shift caused by an IS*256*-associated deletion of a part of γSaβ, the 1a-to-1c shift caused by the insertion of a 14.5 kb IS*Sau5*-associated segment of unknown origin, the 1a-to-1d shift caused by the deletion of phiTDC2, the 1a-to-1g shift caused by the deletion of phiTDC1 and phiTDC2, and the 1a-to-1h shift caused by the IS*256*-mediated integration of pTDC02). Considering the phylogenetic relationships of the 133 isolates, type 1e appears to be the ancestral type, and the 1a-to-1e shift should be regarded as the 1e-to-1a shift caused by the integration of phiTDC1. Supporting this notion, representative strains of the SWP clone did not contain phiTDC1, as mentioned before. The presence of type 1e isolates in SL4 and SL5 can be explained by the deletion of phiTDC1. Importantly, MGEs (prophages, GIs, and IS elements) were involved in PFGE pattern changes in all cases, indicating that MGEs were the drivers of the PFGE pattern changes. It was also noteworthy that all observed changes were explained by one or two genetic events.

### Dynamic changes in the plasmid repertoire during spread in the community

**Fig. 4.**
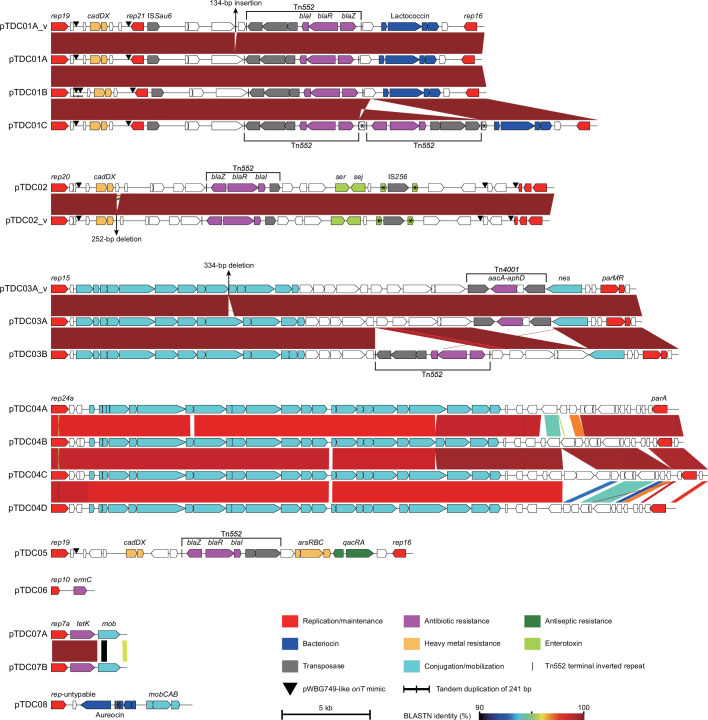
Eight plasmids and their variants found in the TDC clone. The genetic structures of eight plasmids (pTDC01 to pTDC08) and their variants are shown. Replicon types identified by PlasmidFinder and the positions of pWBG749-like *oriT* mimic sequences are indicated. Nucleotide sequence similarities between plasmids are indicated by coloured shading. Notably, a 241 bp sequence containing a pWBG749-like *oriT* mimic sequence was duplicated in tandem in pTDC01B.

As notable alterations in prophages and GIs occurred in the TDC clone during its spread in the community, we next analysed the variation in plasmid repertoire among the 133 isolates. By searching for circular sequences and nonchromosomal contigs in the genome assemblies of all the isolates, we first identified 13 plasmids (corresponding to pTDC01A, pTDC02, pTDC03A, pTDC03B, pTDC04A, pTDC04B, pTDC04C, pTDC04D, pTDC05, pTDC06, pTDC07A, pTDC07B, and pTDC08 in the final plasmid set). The 13 plasmids were classified into eight types (pTDC01-pTDC08) according to their replicon types (Fig. 4 and Table S8). Among these, pTDC02 corresponded to the plasmid found in the C005H04R reference genome. By following Illumina read mapping using the representative sequences of eight plasmids as references and gap closing by PCR, we identified two variants of pTDC01 (pTDC01B and pTDC01C). Thus, structural variations were found in five of the eight plasmids (excluding pTDC05, pTDC06, and pTDC08), which were generated by insertion or deletion of transposable elements (IS*256*, Tn*552*, and Tn*4001*), replacement of small segments, and deletion of short sequences (134–334 bp). In Fig. 4 and Table S8, these variants are indicated by the addition of A, B, C, or D at the end of the plasmid name, and those with very small variations are indicated by the addition of ‘_v’.

Analysis of the distribution of these plasmids among the 133 isolates revealed dynamic changes in the plasmid repertoire during the spread of the TDC clone in the community. Among the eight plasmids, pTDC01 was nearly identical to the pTCH60 plasmid carried by strain TCH60, which is an SWP clone. In addition, the presence of pTDC01-like plasmids in many strains belonging to the SWP clone was suggested by the blast search for the three types of replicons found in pTDC01 (Fig. S5), indicating that pTDC01-like plasmids are well conserved in the SWP clone. Consistent with this, pTDC01 was present in all SL1 and SL3 isolates and one SL5 isolate (C072H50N) that was separated early from the other SL5 isolates (Fig. 3). On the other hand, most SL5 isolates carried pTDC02, a pIB485 family plasmid [9]. These findings indicated that the ancestor of the TDC clone contained pTDC01, and this plasmid was replaced by pTDC02 in SL5 at the early stage of its diversification. Among the six remaining plasmids, while pTDC05 and pTDC08 were present only in isolates from a single household or a single individual, the other four plasmids were found in multiple sublineages (Fig. 3). This distribution pattern suggested that the acquisition of these four plasmids, particularly that of pTDC03 and pTDC04, occurred repeatedly during the spread of the TDC clone.

Of the eight plasmids, six carried AMR genes: the *blaZ* gene on Tn*552* [61] in pTDC01, pTDC02, and pTDC05; the *aacA-aphD* gene on Tn*4001* [62] in pTDC03; the *ermC* gene in pTDC06; and the *tetK* gene in pTDC07 (Fig. 4 and Table S8). Accordingly, the isolates that acquired pTDC03 and pTDC06 became resistant to aminoglycoside (kanamycin and gentamycin) and macrolide (erythromycin), respectively (Fig. 3; see Table S9 for the AMR profile of each isolate). The acquisition of the *blaZ* and *tetK* genes did not change the AMR profiles of the isolates, likely because the TDC clone was already highly resistant to β-lactams because of the acquisition of SCC*mec* and the introduction of *tetK* reportedly does not affect susceptibility to minocycline [63]. In addition to these AMR genes, various other genes that could confer novel phenotypes to each isolate were carried by these plasmids, including genes for potential virulence factors (two minor enterotoxin genes, *ser* and *sej*, by pTDC02), resistance to heavy metals (the *cadDX* operon by pTDC01, pTDC02, and pTDC05 and the *arsRBC* operon [64] by pTDC05) and antiseptics (*qacRA* genes by pTDC05), and bacteriocin synthesis (lactococcin by pTDC01 and aureocin by pTDC08).

Consistent with the relatively short-term acquisition of these plasmids by the TDC clone, seven of the eight plasmids appeared to be either self-transmissible or mobilizable. First, pTDC03 and pTDC04 belong to the pGO1/pSK41 family and pWBG749 family of conjugative plasmids, respectively [9,44], and contain a full set of genes required for conjugational transfer. Second, two small plasmids, pTDC07 and pTDC08, contained *mob* genes; thus, they are apparently mobilizable. Third, pTDC01, pTDC02, and pTDC05 contained one or more sequences corresponding to the ‘pWBG749-like *oriT* mimic’. Thus, they are likely mobilized by pWBG749 family conjugative plasmids via the relaxase–in trans mechanism [44,65, 66]. Moreover, consistent with the transmissibility of these plasmids, many plasmids nearly identical (>99 % sequence identity and >90 % coverage) or very similar (>95 % identity and >80 % coverage) to these plasmids were found in multiple *S. aureus* lineages, even in different *Staphylococcus* species (in the cases of pTDC05 and pTDC08) according to a Megablast search against the NCBI database, with the exception of pTDC01-like plasmids, which were found in very few *S. aureus* lineages (Table S10). Although pTDC06, a small rolling circle replication plasmid belonging to the pT181 family [9], contains no genes/sequences required for transmission, plasmids nearly identical or very similar to pTDC06 were also found in various *S. aureus* lineages and different *Staphylococcus* species (Table S10). Thus, the TDC clone probably had frequent opportunities to acquire these plasmids from other staphylococcal strains during their circulation in the community. This may also explain why plasmids carrying aminoglycoside and macrolide resistance genes were acquired sporadically by the TDC clone despite the lack of administration of these antimicrobials for the treatment/decolonization of the TDC clone in the D clinic.

A somewhat strange observation regarding the plasmid distribution among the 133 isolates was that the introduction of pTDC03 appeared to induce the deletion of pTDC01 and pTDC02 even though they did not share the same replicons (Fig. 3). We therefore performed conjugation experiments from isolate C082H60S-n carrying pTDC03 (encoding an aminoglycoside resistance gene) to two recipients (spontaneous RIF-resistant mutants of the C001H01B isolate carrying pTDC01 and the C006H05R isolate carrying pTDC02). In this set of experiments, although the conjugation efficiency differed between the recipients (3.34×10^−4^ for the pTDC01-carrying recipient and 1.92×10^−7^ for the pTDC02-carrying recipient), transconjugants of pTDC03 were successfully obtained from both recipients, confirming the self-transmissibility of pTDC03 (Fig. S6a). Analyses of the transconjugants from the pTDC02-carrying recipient revealed that two of the 30 analysed transconjugants contained both plasmids, but pTDC02 was lost in the remaining 28 transconjugants (Fig. S6b). This result indicates that pTDC02 and pTDC03 can coexist, but in the absence of appropriate selection, one plasmid (pTDC02 in this case) is easily deleted. In contrast, all transconjugants from the pTDC01-carrying recipient (30/30) contained both pTDC03 and pTDC01 (Fig. S6b). Thus, it appears that pTDC01 can be stably maintained in pTDC03-containing cells *in vitro*. This was a rather unexpected result. It may be too costly and thus not easy for strains to maintain both plasmids for a long time *in vivo*, resulting in the deletion of either plasmid (pTDC01 in this case). It is also possible that some *in vivo* conditions favour (select) pTDC03. The small difference in stability between the plasmids could also result in the deletion of the less stable plasmid. Although we could not determine whether pTDC01 and pTDC02 can coexist, similar events might have induced the replacement of pTDC01 by pTDC02 in the SL5 sublineage.

### Amplification of IS*256* introduced by pTDC02

**Fig. 5.**
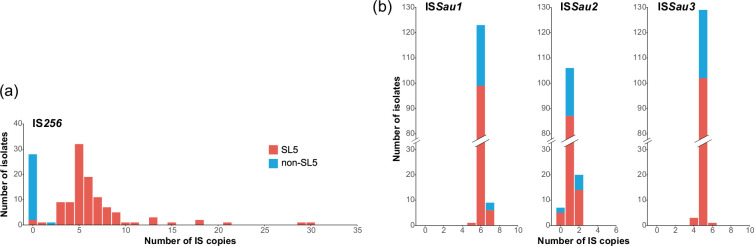
Variations in IS elements among the TDC clone isolates. The copy numbers of the five IS elements found in isolate C005H04R (except for ΨIS*1272* on SCC*mec*) were estimated using ISseeker. All 133 isolates contained one copy of IS*431mec* (data not shown). The variation in the copy number of IS*256* (**a**) was notable compared with that of the other three IS elements (**b**).

As IS elements were also found to be involved in PFGE changes and structural changes in plasmids, the variation in IS elements in the TDC clone was analysed by estimating the numbers of IS copies for each of the five IS elements found in isolate C005H04R (excluding ΨIS*1272*, a remnant of which was found in SCC*mec*) using the ISseeker programme. As shown in Fig. 5, while notable variations in copy number were not detected for the four IS elements other than IS*256*, the number of IS*256* copies was highly variable between the isolates, ranging from 0 to 30 (note that these copy numbers were not accurate due to the limitation of counting IS elements from short-read *de novo* assemblies although the copy numbers of IS*256* in the short-read *de novo* assemblies of C005H04R and C089H64Rb were the same as those in their finished genomes). Interestingly, IS*256* was distributed almost exclusively in the SL5 sublineage (Figs3  5), and its distribution was very consistent with the distribution of pTDC02, which contained an intact IS*256* copy (Fig. 4). This finding suggested that IS*256* was introduced by pTDC02 into SL5 and was variably amplified among the SL5 isolates. Although IS*256* was detected in one isolate outside of SL5, this isolate likely acquired IS*256* independently. In contrast, the four IS elements other than IS*256* were most likely present in the common ancestor and stably maintained in the TDC clone, suggesting that their transposition activities were inhibited by some mechanism(s).

### Alteration of the repertoire of genes encoding potential virulence factors

Finally, we analysed the variation in the virulence-related gene repertoire of the TDC clone. For this purpose, we first identified the virulence-related genes of the reference isolate C005H04R using the VFDB database and then analysed the presence/absence of each gene in all other isolates (Table S11). Of the genes identified in C005H04R, the *lukSF* operon for PVL was carried by phiTDC2, and the innate immunity evasion cluster, which encodes staphylokinase, the chemotaxis inhibitory protein of *S. aureus* (CHIPS), and the staphylococcal complement inhibitor (SCIN), was carried by phiTDC3. As both prophages were well conserved in the TDC clone, these genes were conserved in the TDC clone, except for the emergence of two PVL-negative isolates by the deletion of phiTDC2. The distribution of two enterotoxin genes (*ser* and *sej*) encoded by the pTDC02 plasmid was concordant with the presence of this plasmid. As the γSaα and γSaβ GIs were also well conserved in the TDC clone, the genes located on these GIs [genes for multiple staphylococcal superantigen-like proteins on γSaα and genes for multiple enterotoxins/enterotoxin-like proteins, multiple serine proteases and the second hyaluronate lyase (*hysB*)] [67] were conserved in the TDC clone, except for the PFGE type 1b isolate, in which an IS*256*-mediated deletion of a part of γSaβ occurred. Notably, although several genes were inactivated or deleted in only five isolates, IS*256* was involved in three of the five cases: IS*256* insertion in s*eln* and *selo* on γSaβ each in one isolate and the deletion of *splCDEF, seg*, *selu,* and *seln* by the abovementioned IS*256*-mediated partial deletion of γSaβ (Table S11).

Among the chromosome backbone-encoded genes, four showed in-frame deletions, such as *ebhAB* (encoding a fibrinogen binding protein) and *eap* (encoding an extracellular adherence protein), in several isolates, but these genes appeared to be functionally intact because these deletions occurred at intramolecular repeats. Gene inactivation or deletion was observed for three genes in seven isolates. Three of these isolates from the same patient contained the same nonsense mutation in the *ebhB* gene. The *efb* gene for a fibrinogen binding protein was inactivated by IS*256* insertion in two isolates from the same patient or lost by an IS*256*-mediated deletion in one isolate. In the remaining isolate, the *icaC* gene for intercellular adhesion was also inactivated by the IS*256* insertion.

These results indicate that the variation in the repertoire of virulence-related genes was generated through a relatively small number of genes in limited isolates, and most of the changes were associated with alterations in MGEs and the transposition of IS*256*.

### Conclusion

Through WGS-based analysis of a suspected case of a maternal clinic-centred regional outbreak of CA-MRSA infection, which we previously identified by a PFGE-based analysis, we accurately defined 133 CC30 MRSA isolates as outbreak-related isolates (collectively called the TDC clone), which belonged to the CA-MRSA lineage known as the ST30-MRSA IV or SWP clone. A high-resolution phylogenetic analysis of these isolates revealed that the TDC clone was already present and circulating in the community of this region before outbreak detection, and only the isolates belonging to two sublineages (SL4 and SL5) were directly involved in the outbreak. The long persistence of the TDC clone in multiple individuals and frequent intrahousehold transmission were also revealed by this phylogenetic analysis. Moreover, detailed analyses of the genomic variations revealed that most variations were associated with alterations in MGEs. Changes in the PFGE pattern were generated by alterations in prophages and GIs or IS-mediated insertion of a plasmid or a sequence of unknown origin. Dynamic changes in plasmid content, which were linked to changes in AMR profiles in specific isolates, were induced by the frequent gain and loss of plasmids, most of which were self-transmissible or mobilizable. The introduction of IS*256* by a plasmid (pTDC02) into the SL5 sublineage led to SL5-specific amplification of IS*256,* and amplified IS*256* elements were involved in some of the structural changes in chromosomes and plasmids and generated variations in the repertoire of virulence-related genes in limited isolates. These data revealed how MRSA genomes change during transmission in the community and how MGEs are involved in this process.

## supplementary material

10.1099/mgen.0.001272 Uncited Supplementary Material 1.

10.1099/mgen.0.001272 Uncited Supplementary Material 2.

## Abbreviations

AMR: antimicrobial resistance
BD: Becton, Dickinson and Company
BWA: Burrows-Wheeler Aligner
CA-MRSA: community-associated methicillin-resistant *Staphylococcus aureus*
CC: clonal complex
CFX: cefoxitin
CHIPS: chemotaxis inhibitory protein of *S. aureus*
CLSI: Clinical and Laboratory Standards Institute
EM: erythromycin
FOM: fosfomycin
GI: genomic island
GM: gentamicin
GTR: general time-reversible
IGV: Integrative Genomics Viewer
InDel: insertion/deletion
IS: insertion sequence
KM: kanamycin
LVFX: levofloxacin
MGE: mobile genetic element
MIC: minimum inhibitory concentration
MINO: minocyclcin
ML: maximum-likelihood
MLST: multi-locus sequence typing
MPIPC: oxacillin
NBRP: National Bioresource Project
NCBI: National Center for Biotechnology Information
NJ: neighbor-joining
ONT: Oxford Nanopore Technologies
PBS: phosphate buffered saline
PCG: penicillin G
PCR: polymerase chain reaction
PFGE: pulsed-field gel electrophoresis
PVL: Panton-Valentine leucocidin
RIF: rifampicin
SaPI: *Staphylococcus aureus* pathogenicity island
SCC*mec*: staphylococcal cassette chromosome *mec*
SCIN: staphylococcal complement inhibitor
SL: sublineage
SNP: single nucleotide polymorphism
spa: staphylococcal protein A
SRA: Sequence Read Archive
SRST2: Short Read Sequence Typing for Bacterial Pathogens
SSTI: skin and soft tissue infection
ST: sulfamethoxazole-trimethoprim
ST: sequence type
SWP: South West Pacific
TDC: Tajiri Dermatology Clinic
Tn: transposon
TSB: tryptic soy broth
VCM: vancomycin
VFDB: Virulence Factor Database
WGS: whole-genome sequence
