# Supplementary material for: Mobile genetic element-driven genomic changes in a community-associated methicillin-resistant Staphylococcus aureus clone during its transmission in a regional community outbreak in Japan
Source: Microb Genom. 2024 Jul 17;10(7):001272. doi: 10.1099/mgen.0.001272 (PMC11316552; doi:10.1099/mgen.0.001272)
Supplement: Uncited Supplementary Material 1. [file mgen-10-01272-s001.pdf]

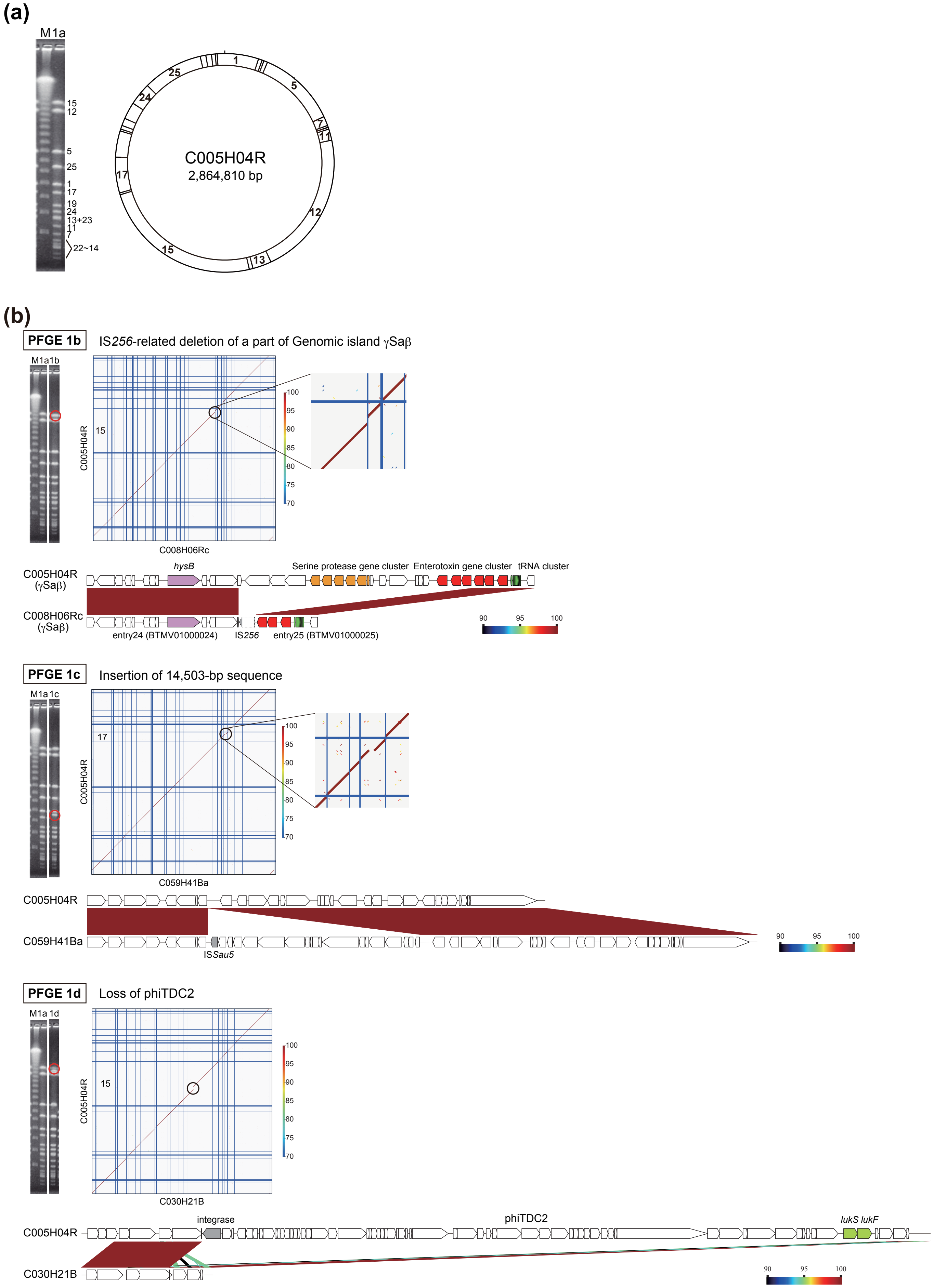


Fig. S1 (continued)

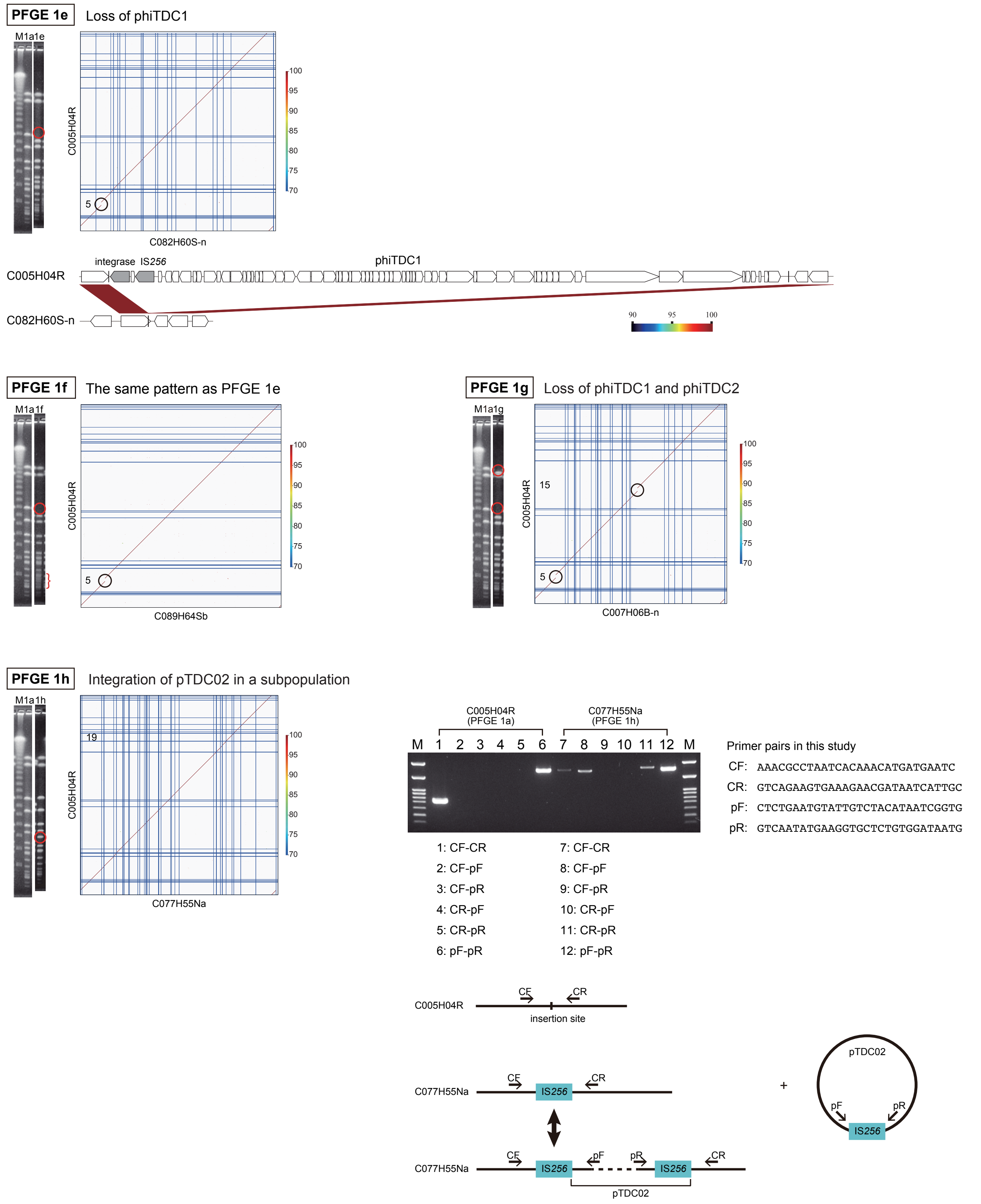


**Fig. S1 The genetic events that generated the variations in PFGE band patterns.**

(a) The SmaI restriction map of the C005H04R chromosome and its PFGE band pattern (type 1a) are shown. M, Lambda PFGE ladder. The positions of the fragments shown in the map are indicated in the left panel for the PFGE band pattern.

(b) The chromosomal regions responsible for the differences in the PFGE band pattern (type 1b-type 1h) and the genetic events that occurred in each region are shown. These regions were identified by aligning assembled sequences of each type to the 29 SmaI fragments of C005H04R and mapping Illumina reads to the C005H04R sequence. In the case of PFGE type 1h, neither alignment nor mapping analysis revealed the responsible genomic difference, but we found that the IS256-mediated integration of pTDC02 was responsible for the 1a-to-1h change, as confirmed by the results of a series of PCR analyses shown in the panel of PFGE 1h (M, 100 bp DNA ladder).

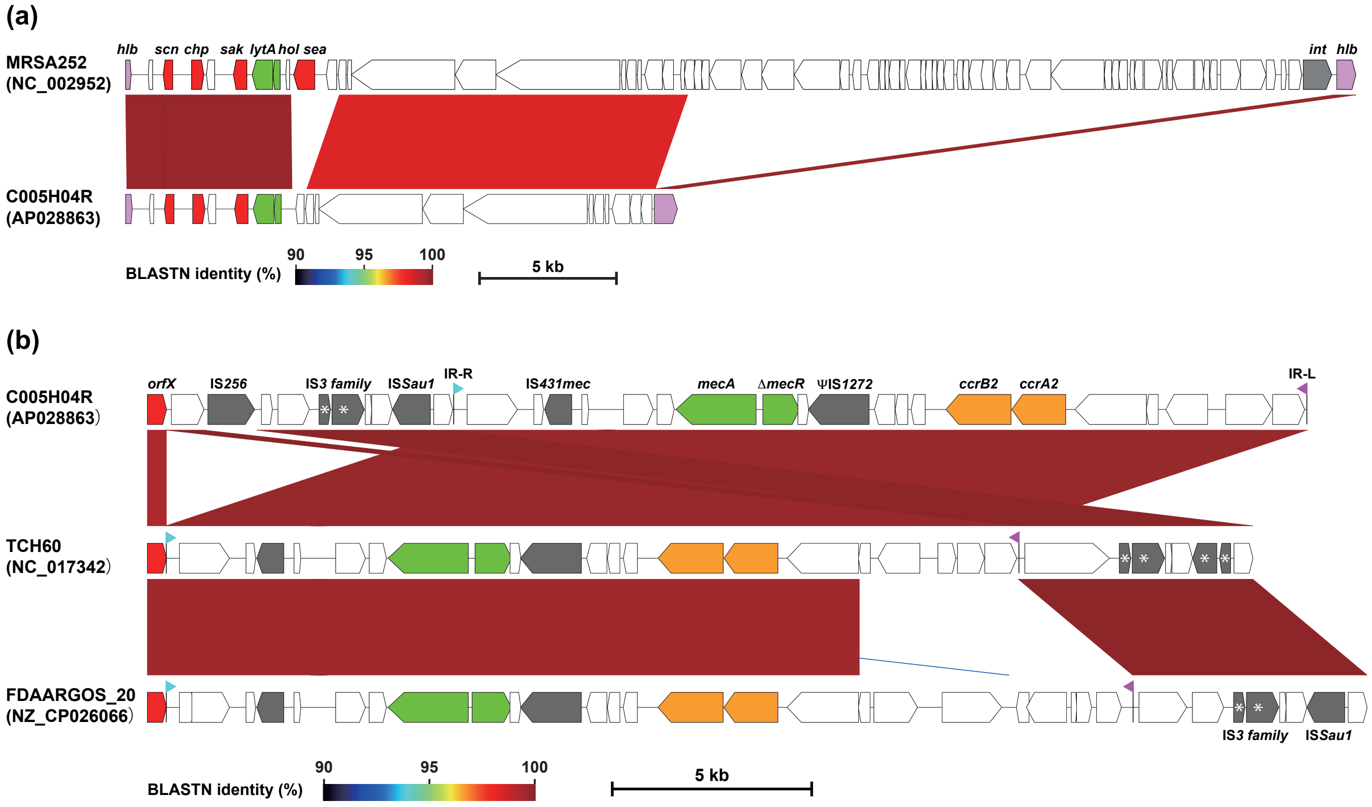


**Fig. S2. Comparison of phiSa3 and SCCmec between the C005H04R isolate and representative CC30 S. aureus strains**
(a) Comparison of phiSa3 between C005H04R and MRSA252 belonging to ST36. (b) Comparison of SCCmec between C005H04R and two strains (TCH60 and FDAARGOS_20) belonging to the SWP clone.

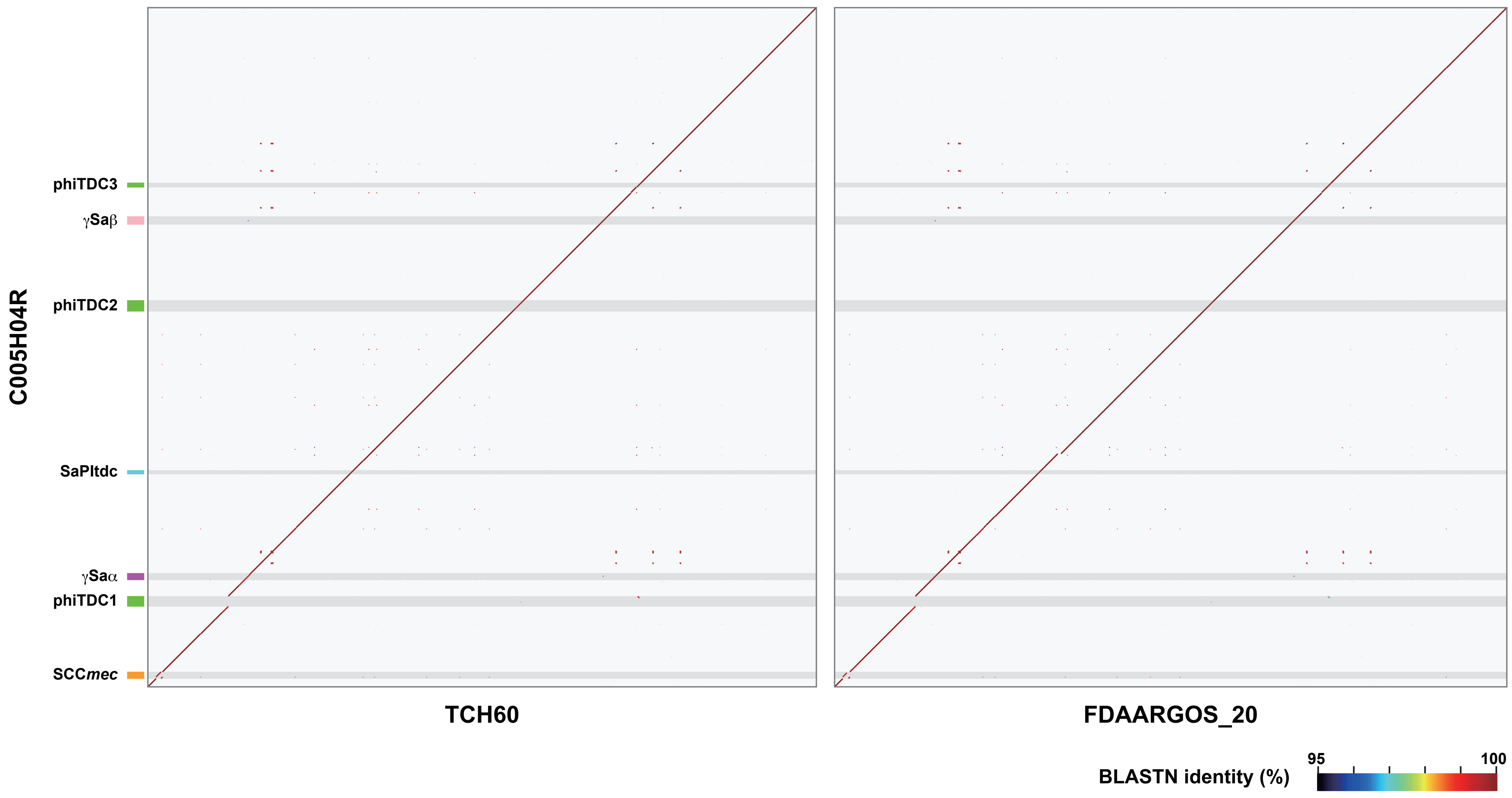


**Fig. S3. Dot plot representation of the chromosome sequence homology between C005H04R, TCH60 and FDAARGOS_20**

The chromosome of C005H04R was almost identical to those of two strains (TCH60 and FDAARGOS_20) belonging to the SWP clone except for a variation in SCCmec and the presence of phiTDC1.

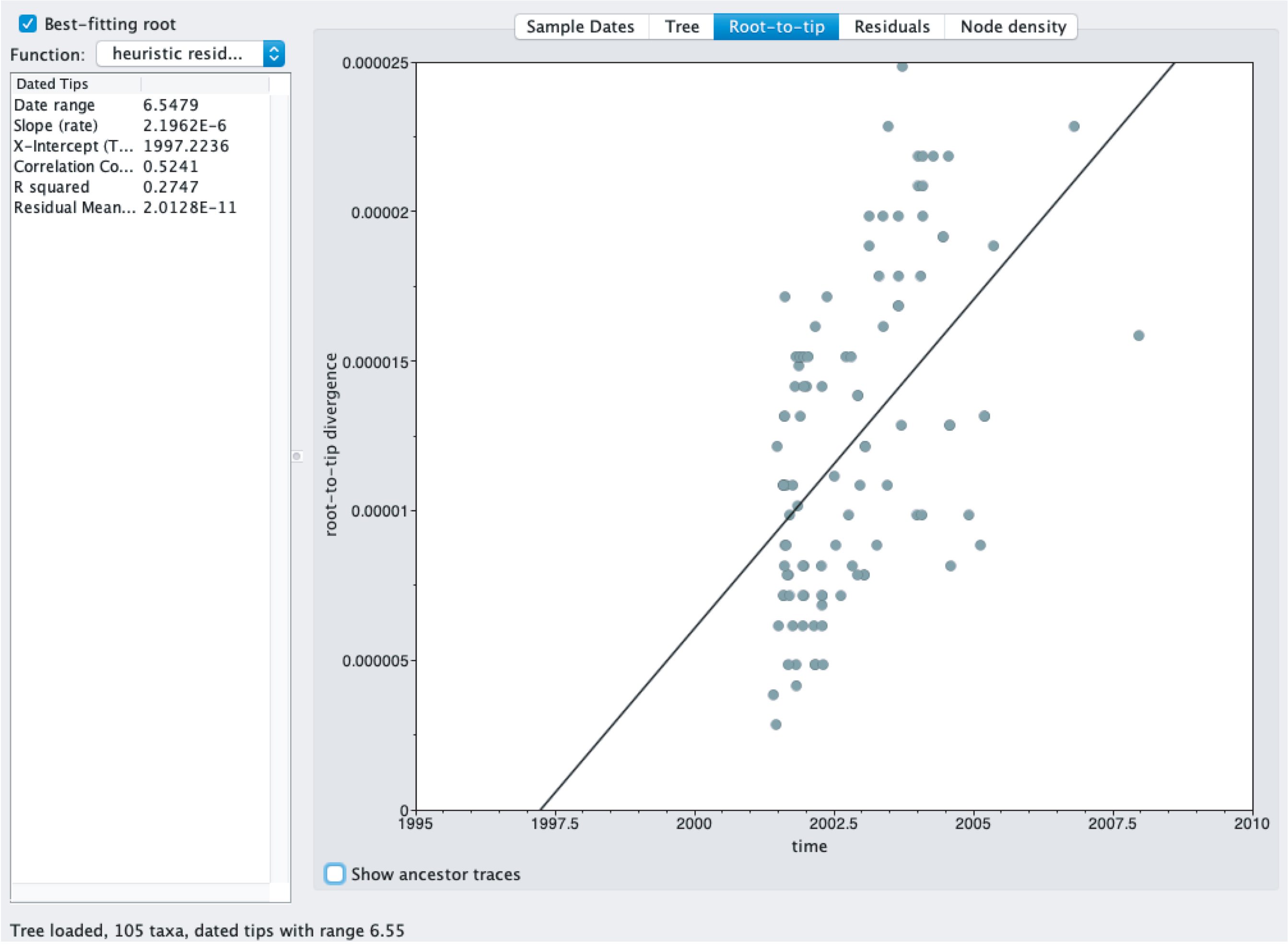


**Fig. S4. Temporal analysis of the TDC clone isolates using TempEst.**
The linear correlation between isolation year and root-to-tip distance from the maximum-likelihood phylogeny for 105 TDC clone isolates was determined using TempEst v1.5.1. Note that, as there were several groups of isolates sharing an identical core genome, the earliest-collected isolates in each group were used in this analysis. Therefore, 105 isolates were included in this analysis.

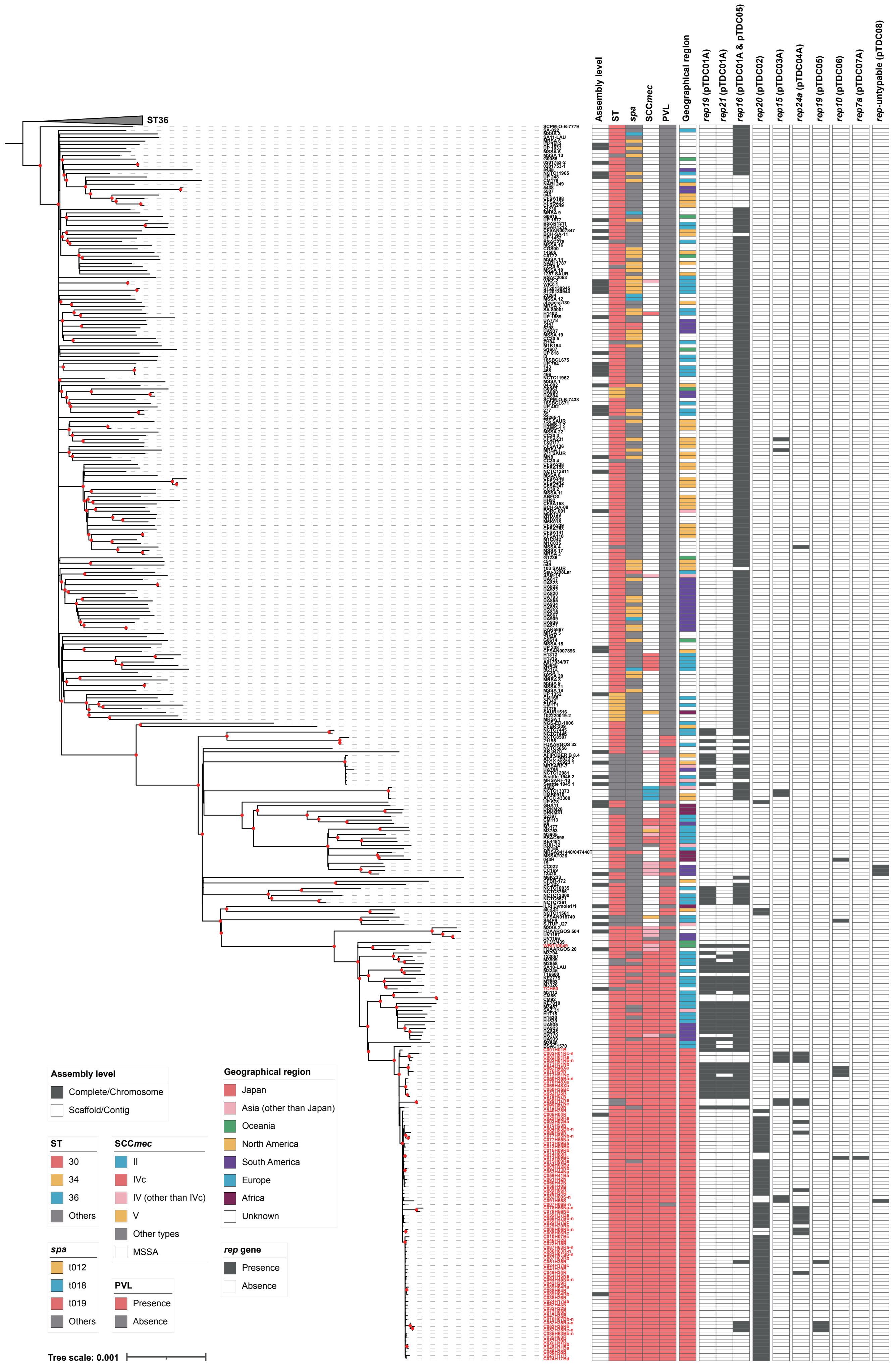


**Fig. S5. The phylogenetic position of the TDC clone in the global population of CC30 *S. aureus* strains and the distribution of replicon sequences identified in the eight plasmids found in the TDC clone**

A midpoint-rooted ML tree was constructed using the same set of data used for Fig. 2 in the main text, and the distribution of replicon sequences identified in the eight plasmids (pTDC01-pTDC08) was mapped to the tree. Nodes showing >80% bootstrap values are indicated by small red circles. The *rep16* sequences of pTDC01 and pTDC05 were indistinguishable. However, the coexistence of *rep19*, *rep21* and *rep16* in many of the strains on the branch that included representative strains of the SWP clone suggests that pTDC01-like plasmids are distributed in the SWP clone.

(a)

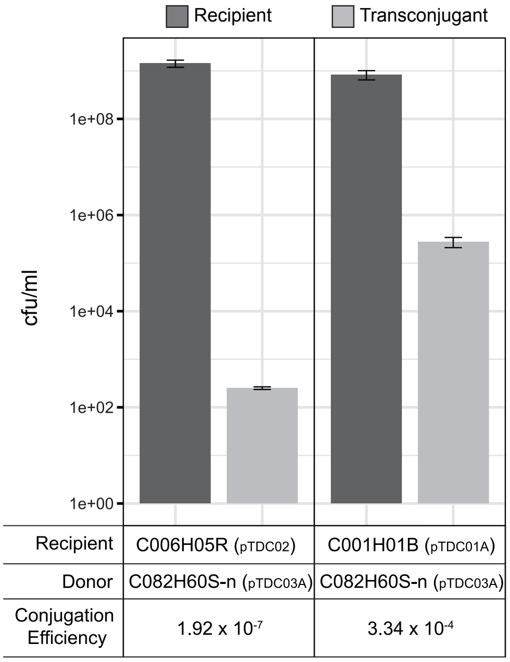


| | | |
|---|---|---|
| Recipient | C006H05R (pTDC02) | C001H01B (pTDC01A) |
| Donor | C082H60S-n (pTDC03A) | C082H60S-n (pTDC03A) |
| Conjugation Efficiency | $1.92 \times 10^{-7}$ | $3.34 \times 10^{-4}$ |

(b)

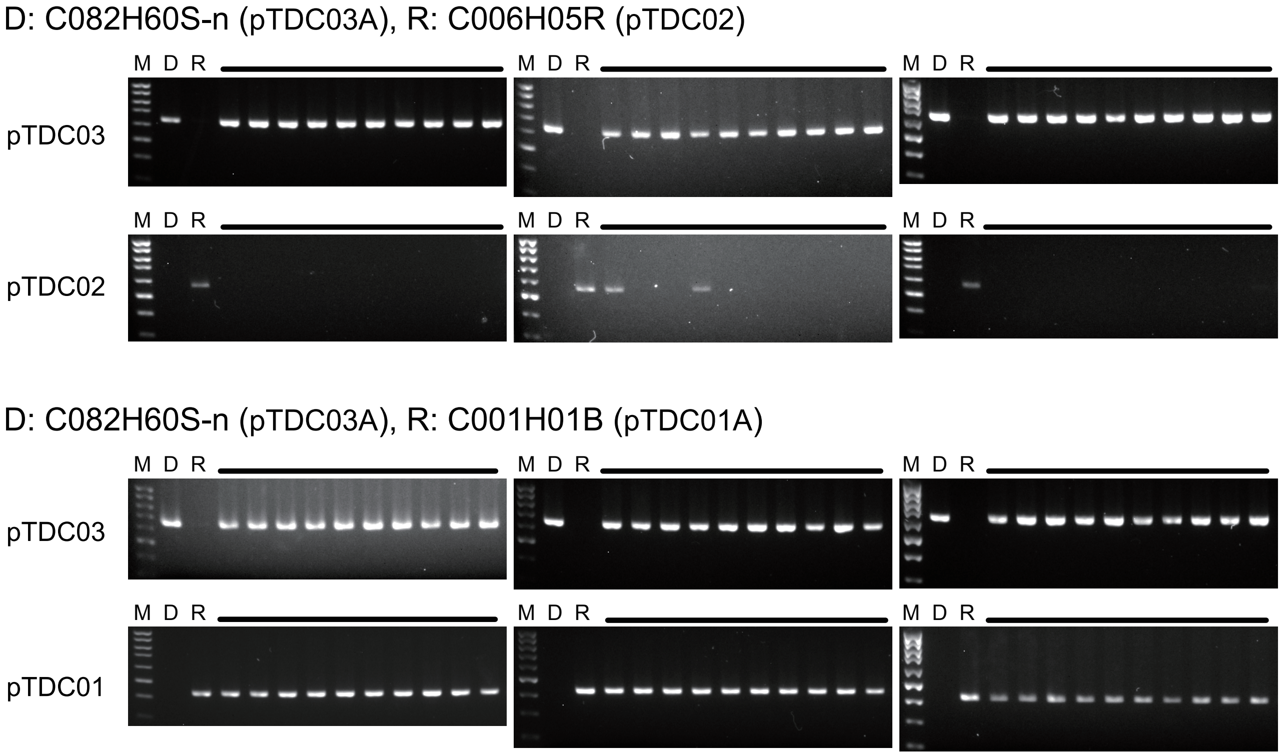


**Fig. S6. Conjugal transfer of pTDC03 to isolates harbouring pTDC01 or pTDC02**

(a) Conjugation efficiencies of pTDC03 from isolate C082H60S-n (donor) to two recipients, rifampicin (RIF)-resistant mutants of isolates C006H05R and C001H01B harbouring pTDC02 and pTDC01A, respectively, are shown.
The error bars represent the standard deviations of three biological replicates from three independent experiments.
Note that pTDC03 encodes an aminoglycoside resistance gene.
(b) PCR detection of each plasmid in single colonies of transconjugants. M; size marker, D; donor strain, R; recipient strain.
